# Osteochondral Peg Fixation for Chondral Fragment of the Knee in Adolescent Patients: A Report of Two Cases

**DOI:** 10.1155/2021/9958012

**Published:** 2021-07-08

**Authors:** Katsuhiro Ichikawa, Hiroyasu Ogawa, Kazu Matsumoto, Haruhiko Akiyama

**Affiliations:** ^1^Department of Orthopaedic Surgery, Gifu Prefectural General Medical Center, Noishiki 4-6-1, Gifu, Gifu, Japan; ^2^Department of Orthopaedic Surgery, Ogaki Tokushukai Hospital, Hayashi-machi 6-85-1, Ogaki, Gifu, Japan; ^3^Department of Orthopaedic Surgery, Gifu University Graduate School of Medicine, Yanagido 1-1, Gifu, Gifu, Japan

## Abstract

**Introduction:**

Purely chondral injuries of the knee are relatively rare, and no consensus exists on the appropriate treatment in such cases. We describe two adolescent patients with chondral injury of the knee who were successfully treated by osteochondral peg fixation. *Patients, Concerns, and Clinical Findings*. In case 1, a 14-year-old boy presented with complaints of right knee pain after landing on his leg while playing basketball. Radiography and computerized tomography revealed no abnormalities. However, magnetic resonance imaging revealed a chondral defect in his lateral femoral condyle and a loose chondral fragment measuring 6.5 cm^2^. In case 2, a 12-year-old boy presented with complaints of left knee pain after a rotational injury while playing baseball. Similar to case 1, magnetic resonance imaging revealed a chondral defect in his lateral femoral condyle and a loose chondral fragment measuring 3.0 cm^2^. *Primary Diagnosis, Interventions, and Outcomes*. The two patients were treated by surgical fixation using osteochondral pegs, which were harvested from the femoral condyle. After a year, postoperative computerized tomography and magnetic resonance imaging showed union of the chondral fragment with the osteochondral pegs and surrounding tissue. In both cases, the Lysholm score was 100 points at the final follow-up more than 2 years after surgery.

**Conclusion:**

The findings reported herein suggest that osteochondral peg fixation is a feasible treatment option for chondral injury of the knee, with satisfactory outcomes.

## 1. Introduction

Pure chondral injury of the knee is uncommon, and no consensus exists on the optimal treatment of this injury. This injury is typically caused by an acute shear-off trauma, such as dislocation of the patella or twisting injury of the knee joint [1]. Children are more prone to shear-off trauma than adults, owing to the lack of mechanical strength of the bone–cartilage junction [2]. Previous studies suggest that articular cartilage without osseous tissue has limited capacity to heal [3], and articular cartilage defects may contribute to the development of osteoarthritis of the knee in adolescents [4]. Several surgical techniques have been reported for treating pure chondral lesions, including fixation with absorbable screws, suture, bone pegs, fibrin glue, and chondral darts [5–12]. Although they are not employed for cartilage fragment fixation, allograft plug techniques and osteochondral autologous transplantation (OAT) have been used as treatment methods for osteochondritis dissecans [13–17]. The technique involving the use of autogenous osteochondral plugs for the fixation of osteochondral fragments (mosaicplasty) has been reported to be associated with good clinical outcomes [13–15]. However, to our knowledge, there are no previous reports regarding the treatment of these defects via fixation by an osteochondral autograft transplantation combined with microfracture. In this report, we describe the cases of two adolescent patients with a pure chondral injury who were treated by fixation using osteochondral pegs combined with microfracture, resulting in satisfactory clinical outcomes. This report was approved by the institutional review board of our institution.

## 2. Case Presentation

### 2.1. Case 1

**Figure 1:**
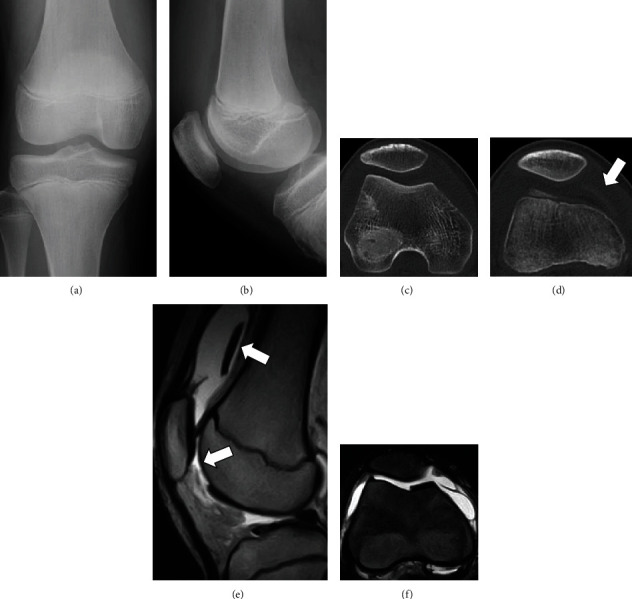
Representative images of case 1. Preoperative anteroposterior (a) and lateral (b) radiography and axial computerized tomography of the knee showing no abnormality (c) and a chondral fragment with no bony element (d). Preoperative sagittal (e) T2-weighted and axial (f) T2-weighted, fat-suppressed magnetic resonance imaging showing a chondral defect in the lateral femoral condyle and a loose chondral fragment in the medial suprapatellar pouch.

**Figure 2:**
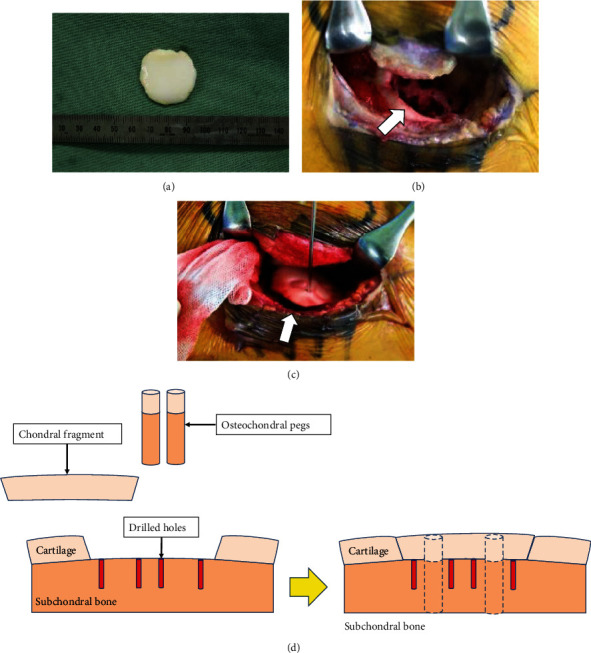
Intraoperative findings of case 1: (a) the chondral fragment measuring 2.5 × 2.6 cm in size, appearing to contain no bony element; (b) the chondral defect at the lateral femoral condyle after the microfracture drilling procedure; (c) fixation of the chondral fragment with two 6 × 13 mm two osteochondral pegs; (d) schematic overview of surgical technique. Initially, the mother floor was drilled by Kirshner wire and then, the fragment was press-fitted by two osteochondral bone pegs which were harvested from the lateral femoral condyle.

**Figure 3:**
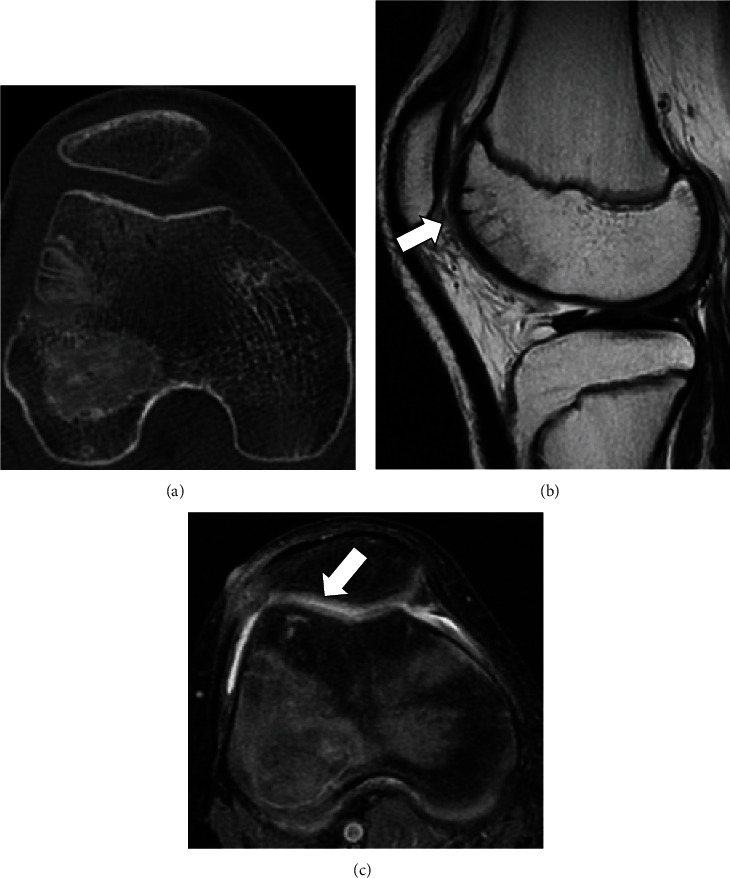
Postoperative axial (a) computerized tomography and sagittal (b) T2-weighted and axial (c) T2-weighted, fat-suppressed magnetic resonance image showing a smooth, healing chondral surface with osteochondral peg in place 2 months after osteochondral peg fixation.

A fourteen-year-old boy injured his right knee upon landing following a jump during basketball practice, and he developed pain and swelling of the right knee. Three days after the injury, he visited a local doctor because the symptoms persisted. Physical examination revealed swelling of his right knee, patella bounce, and tenderness, but no range of motion limitation (0°–140°). No knee instability was observed, and the Lachman test was negative. Radiography showed no abnormal findings (Figures 1(a) and 1(b)). Computerized tomography (CT) revealed no bony abnormalities and a chondral fragment without bony elements (Figures 1(c) and 1(d)). Magnetic resonance imaging (MRI) of the right knee showed a chondral defect in his lateral femoral condyle and a loose chondral fragment in the medial suprapatellar pouch (Figures 1(e) and 1(f)). Five days after the injury, he was referred to our hospital for further treatment. Based on the clinical findings and imaging studies, we diagnosed a pure detached chondral fracture of the lateral femoral condyle and planned a repair of the chondral fracture using osteochondral pegs through osteochondral autograft transfer system (OATS; Arthrex, Naples, FL) under general anesthesia. Arthroscopic exploration confirmed the MRI findings, with visualization of a large detached chondral fragment and a defect located on the anterior aspect of the lateral femoral condyle. No other intra-articular injury was observed. Subsequently, the knee joint was opened by a lateral parapatellar approach, and we extracted a loose chondral fragment found in the suprapatellar pouch. The size of the fragment was 2.5 cm × 2.6 cm, and it appeared to have no bony tissue (Figure 2(a)). We found the defect in the anterior aspect of the lateral femoral condyle, which corresponded to the size of the chondral fragment, and drill holes, along with 1.6 mm Kirshner wire, were introduced to promote repair of the chondral layer at the site through bone marrow stimulation (Figure 2(b)). The chondral fragment was anatomically refixed using two osteochondral bone pegs (6.0 mm in diameter and 13 mm in depth) harvested from the lateral femoral condyle using the OATS (Figures 2(c) and 2(d)). Both pegs exhibited good fixation, with stable borders and good restoration of the articular surface. Four days postoperatively, the patient began passive-assisted mobilization of the knee, followed by active mobilization after three weeks. Partial weight-bearing at one-third of the body weight was allowed three weeks postoperatively, and full weight-bearing was allowed at six weeks postoperatively. At two months postoperatively, radiography, CT, and MRI scans confirmed the union of the chondral fragment and osteochondral peg, with smooth continuity of the articular surface (Figure 3). The Lysholm score was 100 points, and he returned to sports at 3 months postoperatively with no limitations. He had no symptoms at the final follow-up two years after surgery.

### 2.2. Case 2

**Figure 4:**
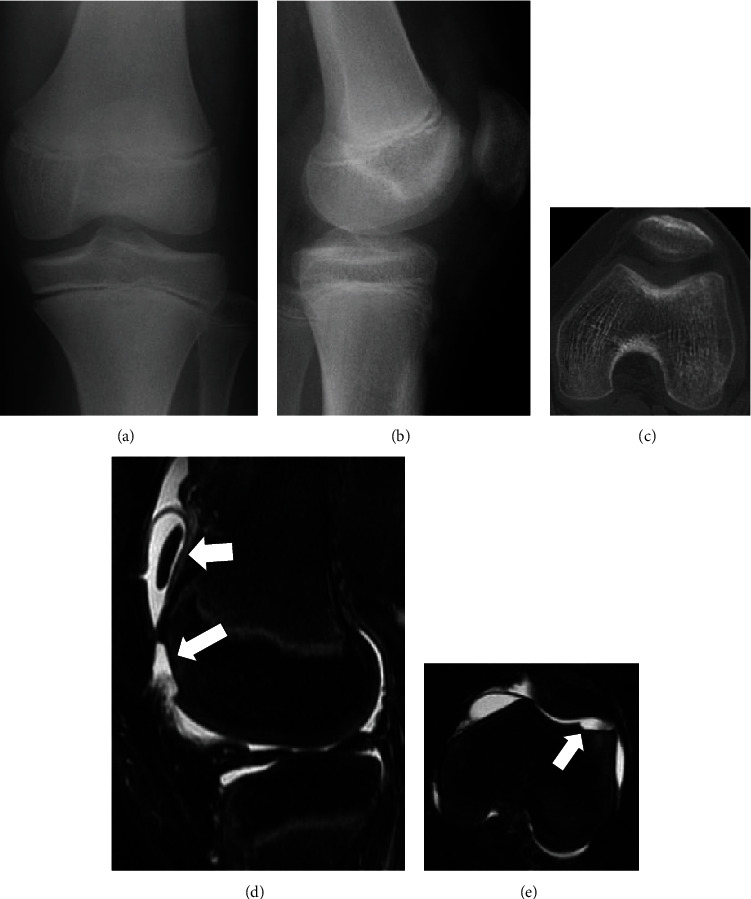
Representative images of case 2. Preoperative anteroposterior (a) and lateral (b) radiography and axial computerized tomography (c) of the knee showing no bony injury. Preoperative sagittal (d) and axial (e) T2-weighted, fat-suppressed magnetic resonance imaging showing a chondral defect in the lateral femoral condyle and a loose chondral fragment in the medial suprapatellar pouch.

**Figure 5:**
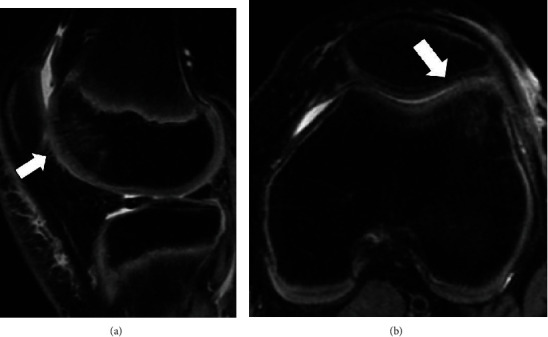
Postoperative sagittal (a) and axial (b) T2-weighted, fat-suppressed magnetic resonance images taken 2 months after osteochondral peg fixation showing a complete bony and chondral union and a continuous articular surface.

A twelve-year-old boy twisted his left knee inwards while running during baseball practice and developed left knee pain. Two days after the injury, he visited a local doctor. Physical examination revealed crepitation during knee flexion, but he had no swelling, patellar bounce, tenderness, or limited range of motion (0°–140°). No instability was noted, and the Lachman test was negative. Radiography and CT scans showed no abnormalities (Figures 4(a) and 4(b)), although MRI of the left knee showed a chondral defect in the lateral femoral condyle and a loose chondral fragment in the medial suprapatellar pouch (Figures 4(c) and 4(d)). Twenty-six days after injury, he presented to our department with no symptoms. We diagnosed a pure detached chondral fracture of the lateral femoral condyle on the basis of his clinical findings and imaging results, and repair of the chondral fracture was performed using osteochondral pegs. During the initial arthroscopy, a large detached chondral fragment was identified, and the defect site, located on the lateral femoral condyle, was inspected. Thereafter, a loose chondral fragment measuring 2 cm × 1.5 cm in size, with no osseous tissue in the suprapatellar pouch, was extracted through a lateral parapatellar arthrotomy. We found the floor of the defect originating in the lateral femoral condyle, which had the same size as that of the chondral fragment, and drill holes were made at the site for bone marrow stimulation. The chondral fragment was anatomically repaired using two 4.75 × 24 mm-sized osteochondral bone pegs harvested from the lateral femoral condyle. Postoperatively, the patient underwent a standard therapy protocol, similar to that described in case 1. At two months, radiography, CT, and MRI studies revealed healing and integration of the fragment with the surrounding cartilage and the osteochondral pegs (Figure 5). He returned to sports activity at three months postoperatively, and the Lysholm score was 100 points at the final follow-up 2 years after surgery.

## 3. Discussion

We described two adolescent patients with pure chondral injury of the knee who was successfully treated by an osteochondral autograft transplantation combined with microfracture. To our knowledge, this is the first report on pure chondral injury treated by fixation using this procedure. Pure chondral injury is rare among knee injuries, and it usually occurs in adolescents, owing to the relatively weak shear strength at the bone–cartilage junction [2]. This injury usually occurs during sports activity, particularly during rotational or twisting motion of the knee, which leads to patellar subluxation or dislocation, causing injury to the lateral femoral condyle or trochlear groove [1]. The articular cartilage has poor healing potential [3], and restoring the articular cartilage of the joint surface is important to prevent the development of knee osteoarthritis in the future [4]. Therefore, surgical treatment is usually required. Surgical treatment of chondral fractures of the knee includes several options, such as fixation using bioabsorbable screws, bone pegs, chondral darts, and fibrin glue [5–12]. Although studies of almost all of these techniques have reported successful outcomes, complications or disadvantages of these methods have also been reported [17–20]. For example, synovitis with effusion and cartilage damage caused by the backing out of pins have been reported as complications of techniques using bioabsorbable screws [18–20], and complications involving delamination of the chondral surface of the plug have been described in cases of fixation using osteochondral allograft plugs [17]. In the present cases, we performed osteochondral peg fixation using OATS, which was originally reported for the treatment of osteochondral dissecans through mosaicplasty [13–15]. The procedure, which involves the implantation of osteochondral plugs through the fragment, was described by Berlet et al. [13], and Miniaci et al. reported good clinical outcomes among twenty patients with osteochondral dissecans who were treated using this technique [14]. This method of fixation for pure chondral injury has some advantages. First, the chondral fragment is fixed in the chondral layer of the osteochondral peg. As a result, there is no difference in the speed of integration between the bone and cartilage tissue, biologically speaking, which contributes to preventing complications such as the backing out of screws postsurgery. Second, osteochondral pegs can be larger in diameter in OATS than in other fixation techniques; hence, the fragment can be rigidly fixed. However, problems related with the donor site are potential disadvantages of OATS, and bone peg fixation, which involves the harvesting of bone pegs from the ipsilateral tibia, may be less invasive than OATS in terms of the donor sites [9, 10]. In our cases, we did not perform biopsies as described in a previous report, so we cannot determine the level of delamination precisely [5, 6]. Nakamura et al. reported a case of an injury in the deep zone in cartilage and integration of the chondral fragment with the subchondral bone after fixation of the chondral fragment [5]. They hypothesized that the bone marrow cells recruited by subchondral curettage participated in healing. In addition, the spindle-shaped cells identified on the surface of the chondral fragment suggested that the stem cells promoted tissue repair in bone marrow stroma by differentiating into chondrogenic and osteogenic cells to restore the osteochondral junction. Thus, we believe that chondral fragments integrate with the subchondral bone, particularly in children. Microfracture procedure combined with osteochondral autograft transplantation also facilitates the integration of chondral fragment with the subchondral bone. Although we confirmed the union of the osteochondral peg and chondral fragment without any problem about donor site morbidity by MRI and both patients had already resumed sports activity prior to the final follow-up, further studies with a large study population and long-term clinical and radiographic follow-up are warranted to confirm the safety and efficacy of the procedure.

To conclude, we described two patients with a pure chondral fracture who were successfully treated by an osteochondral autograft transplantation combined with microfracture. These pure chondral fragments were relatively large; hence, curetting, microfracture, and resurfacing procedures and biologic augmentation were potential treatment options. However, this injury frequently occurs in young and active patients who have high activity levels. Fixation procedures require a shorter period of weight-bearing restrictions and allow for a faster return to sports activity than is observed with other treatments. Therefore, we recommend this procedure as a feasible option for the treatment of pure chondral injuries of the knee.

## Data Availability

The data in this article are available on request to the corresponding author.
